# Isothermal recombinase polymerase amplification based diagnostics for female genital schistosomiasis and human papillomavirus: a review of combined molecular diagnostic opportunities

**DOI:** 10.1017/S0031182025101248

**Published:** 2025-12

**Authors:** Lucy Isabelle Smith, Sanjeev Krishna, Helen Kelly, Amaya Bustinduy, Bonnie Webster

**Affiliations:** 1 Clinical Research Department, London School of Hygiene and Tropical Medicine, London, UK; 2 Department of Medicine, City St George’s University of London, London, UK; 3 Department of Science, Natural History Museum, London, UK; 4 Institut für Tropenmedizin, Universitätsklinikum Tübingen, Tübingen, Germany; 5 School of Public Health, Physiotherapy and Sports Science, University College Dublin, Dublin, Ireland

**Keywords:** female genital schistosomiasis, HPV, isothermals, molecular diagnostics, recombinase polymerase amplification, Schistosoma, sexual health

## Abstract

Women in sub-Saharan Africa face complex, multifaceted challenges to their health, including a high burden of infectious diseases aggravated by socioeconomic factors. Parasitic and sexually transmitted infections both cause significant morbidity and mortality. Co-infections compound these effects, leading to high rates of chronic illness and making diagnosis and treatment challenging. There are no integrated approaches for the detection of female genital schistosomiasis (FGS), a gynaecological condition caused by *Schistosoma haematobium*, and high-risk human papillomavirus (HR-HPV), responsible for over 90% of all cervical cancer cases worldwide. FGS is a chronic condition with health outcomes such as infertility and abortion and remains severely under-reported. HR-HPV infection is the main aetiological agent of cervical cancer, the leading cause of cancer death in women in sub-Saharan Africa. Both can be disabling and stigmatizing to the sufferer. A key to disease management at patient and community levels is accurate and available diagnostics. Due to both FGS and HPV diagnostics utilising cervicovaginal samples, they are ideal candidates for a multiplex molecular diagnostic. The standard molecular diagnostics (namely PCR), through the detection of pathogen DNA, are constrained in low resource settings by requirement of a highly reliable source of energy, reliance on a cold-chain, and prohibitive costs. Isothermal molecular diagnostics are an alternative method to PCR that are more suited to basic settings. This review explores current isothermal diagnostics, with a focus on RPA/RAA, a very simple isothermal technology, for FGS and HPV and proposes the development of a multiplex isothermal diagnostic test to enable integrated screening.

## Graphical abstract

**Figure:**
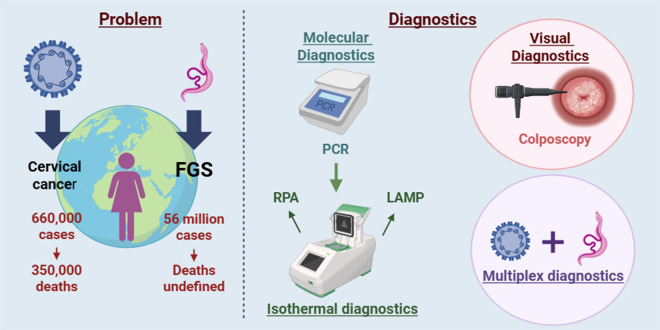

## Introduction

Women in sub-Saharan Africa (SSA) face complex health challenges due to a combination of environmental and socio-economic factors (Habib et al., 2021). Social determinants of health for women may differ between countries that have unique cultures and dominant religions, but women’s health tends to remain universally neglected (Pons-Duran et al., 2019). An ongoing challenge in women’s health in SSA is the presence of co-infections in the genital tract. Co-infections can be drawn from any aetiology – viral, bacterial, fungal, and parasitic.

High-risk human papillomavirus (HR-HPV) infection and female genital schistosomiasis (FGS) due to *Schistosoma haematobium* egg-deposition in the genital tract can exist as co-morbidities among women in SSA and can share clinical manifestations. Both increase the risk of cervical lesions and adverse sexual and reproductive health outcomes by causing chronic inflammation and damage to the genital mucosa (Fernandes et al., 2015; Rossi et al., 2024).

Nucleic acid amplification tests (NAATs) can detect pathogen DNA even in asymptomatic patients (Krishna and Cunnion, 2012). Polymerase chain reaction (PCR) is the most common NAAT that utilizes thermocycling (cycling through set temperatures) to enable DNA amplification and has been used for both diagnosing active FGS and HPV infections. It is highly sensitive and specific, but the high energy needs, infrastructure, and cost can be prohibitive for its use in low-resource settings.

An additional consideration when developing diagnostics for use in low-resource settings is the possibility of multiplexing or detecting multiple pathogens within one assay. This reduces the sample number required from each patient, requires less reagents and consumable plastics, and can reduce the number of visits a patient has to make to the clinic, which may be remote or difficult to access. In part due to its simple primer design, the PCR platform has been used successfully to develop multiplex assays.

Isothermal assays (one constant temperature) enable DNA amplification at a single set temperature, requiring less equipment and infrastructure, making them more acceptable in low resource settings. The most used isothermal platforms are loop-mediated isothermal amplification (LAMP) and recombinase polymerase/recombinase aided amplification (RPA/RAA). LAMP has been used extensively but has complex assay design. Due to its simple assay design, RPA/RAA has high potential to address some of the accessibility issues for NAATs in low-resource settings.

This review explores the available RPA/RAA diagnostic assays for FGS and HPV, and considers their potential for a multiplex diagnostic, with an emphasis on their suitability for use in low-resource settings.

Electronic searches were conducted on MEDLINE, PubMed and EMBASE databases, using combined ‘*Schistosoma haematobium*’ ‘human papillomavirus’ and ‘recombinase polymerase amplification’ search terms from the year 2000 to present day.

## Female genital schistosomiasis

### Female genital schistosomiasis epidemiology and clinical manifestations

Schistosomiasis is a neglected tropical disease (NTD) caused by infection with parasitic trematodes of the *Schistosoma* (*S*) genus, with 700 million people at risk globally (Buonfrate et al., 2025). Schistosomiasis can be broadly divided into two clinical syndromes depending on the location of the adult worms; intestinal schistosomiasis mainly caused by *S. japonicum* and *S. mansoni*, and urogenital schistosomiasis caused by *S. haematobium*. FGS is a morbidity associated with urogenital schistosomiasis caused when eggs of *S. haematobium* become trapped in the female reproductive organs. Chronic disease can result in infertility, ectopic pregnancy, and chronic inflammation (Lamberti et al., 2024).

FGS is mainly associated with urogenital schistosomiasis, which is endemic to SSA, parts of the Middle East, and the southern European island of Corsica (Boissier et al., 2016). It is estimated that between 20 and 56 million women globally are affected by FGS, although challenges in the availability and accuracy of diagnostics make it difficult to make a robust estimate of the global burden (World Health Organisation, 2022).

**Figure 1.**
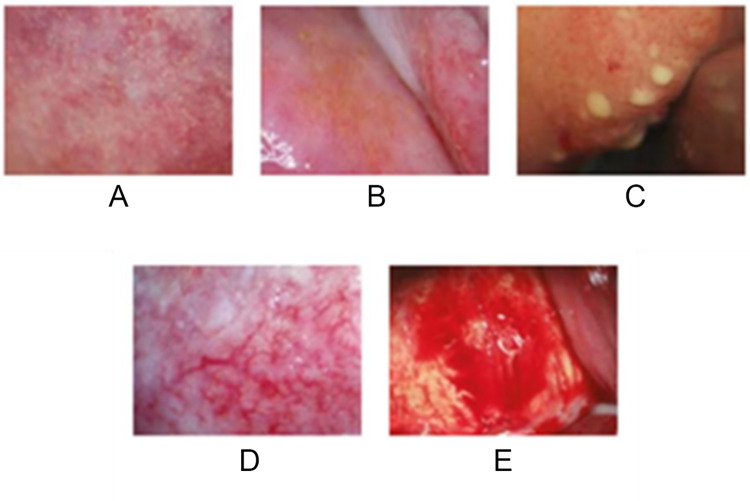
Colposcopy images of characteristic FGS pathology, including (A) Grainy sandy patches; (B) Homogenous yellow patches; (C) Rubbery papules; (D) Abnormal blood vessels; and (E) Severe contact bleeding (Figure adapted from UNAIDS, 2019).

*S. haematobium* eggs are highly antigenic, triggering granuloma formation which may later turn into fibrotic scar tissue which does not resolve even after the egg has been destroyed (Kjetland et al., 2012; see Figure 1). Early symptoms of FGS include unusual vaginal discharge, genital itching, coital pain. and contact bleeding (Bustinduy et al., 2022). The severity of symptoms is linked to the intensity of the *S. haematobium* infections leading to increased egg deposition, which may be exacerbated by reinfection. When left untreated, chronic FGS can lead to sub-fertility, infertility, and an increased risk of ectopic pregnancy even after the *S. haematobium* infection has been cured (Kjetland et al., 2012). Additionally, there is some evidence of association between FGS and cervical pre-cancer (Rafferty et al., 2021; Sturt et al., 2025). The diagnosis of FGS is complex and not readily accessible in most endemic settings (Bustinduy et al., 2022).

One of the major challenges in FGS diagnosis is that symptoms overlap with those associated with sexually transmitted infections (STIs) and other gynaecological conditions (Bustinduy et al., 2022). This can result in women not seeking medical interventions and can impart stigma on the sufferer if misdiagnosed (Engels et al., 2020).

### Female genital schistosomiasis diagnosis

FGS diagnostics vary widely depending on the setting and resources available. The simplest form of FGS diagnosis is based on associated symptoms in combination with the routine diagnosis of *S. haematobium* infections, typically by urinary egg microscopy (Lamberti et al., 2024). However, symptoms are non-specific and urinary egg microscopy has low correlation with genital lesions.

Other diagnostic methods including urine-based NAATs (Cnops et al., 2013) and antigen tests for circulating anodic antigen (CAA; Hoekstra et al., 2021) have been used to confirm infection with *S. haematobium* as a proxy method of FGS risk with unclear link to genital track involvement. More specific methods are needed to accurately diagnose FGS and monitor levels of pathology and associated morbidity (Bustinduy et al., 2022).

The specific diagnosis for FGS currently necessitates either visualizing the cervix directly or taking cervicovaginal samples such as swabs or cervicovaginal lavage (CVL) for *Schistosoma* egg or DNA retrieval (Sturt et al., 2020). In low-resource settings, visualizing the cervix through colposcopy to detect FGS-associated lesions is the most common diagnostic method (Lamberti et al., 2024). However, colposcopy is limited as it requires highly trained personnel to conduct the procedures and identify FGS-associated lesions. Furthermore, visual identification lacks specificity, expert agreement between diagnoses remains poor, and not all cases present with lesions (Bustinduy et al., 2022; Sturt et al., 2023). Current developments in equipment, such as hand-held colposcopes and artificial intelligence-aided diagnostic technology, aim to make colposcopy more accurate and more accessible to women in low-resource areas (Lamberti et al., 2024).

NAATs that specifically detect *S. haematobium* eggs or DNA, typically based on PCR, have been performed using different genital samples including CVL (Kjetland et al., 2009), operator-collected cervical swabs (Ursini et al., 2023), and cervico-vaginal self-swabs (Sturt et al., 2020; Shanaube et al., 2024). Isothermal assays utilizing LAMP and RPA have been developed more recently (Gandasegui et al., 2015; Archer et al., 2022) demonstrating that molecular diagnostic assays can maintain high levels of sensitivity and specificity while being suitable for low-resource settings. While NAATs are likely more specific and sensitive than colposcopy, older lesions where the causative eggs has already degraded will not be DNA-positive, so may be missed if DNA amplification methods are used in isolation. This is especially relevant for FGS as lesions persist even when there is no active *S. haematobium* infection.

### Female genital schistosomiasis treatment and management

Schistosomiasis can be treated with praziquantel, a drug that kills the adult worms in the blood vessels. Distribution of praziquantel by mass drug administration (MDA) is the primary form of control of schistosomiasis in endemic areas. Although people in endemic regions often become re-infected, regular treatment is essential in limiting symptoms and preventing morbidity (Faust et al., 2020).

There is limited evidence from small studies, and only one randomized controlled trial, that indicates that praziquantel has limited action against FGS lesions (Arenholt et al., 2024). Therefore, diagnosis should be coupled with other approaches to case management to improve patient outcomes. With the increasing awareness of FGS, it is now recognized that it is essential that schoolgirls, female adolescents, and women of all ages in *S. haematobium* endemic areas, receive praziquantel treatment regularly to prevent FGS or help reduce the risk of high levels of associated morbidity (Engels et al., 2020; Lamberti et al., 2024).

## Human papillomavirus

### Human papillomavirus epidemiology and clinical manifestations

HPV is one of the most common STIs worldwide; over 80% of sexually active individuals will acquire HPV at least once in their lifetime (Chesson et al., 2014). Up to half of infections clear within 6 months and approximately 90% clear within two years after acquisition (Plummer et al., 2007). Over 200 genotypes have been identified, 40 of which affect the genital tract. HPV genotypes are classified into low-risk (non-oncogenic) and high-risk (oncogenic) types based on their potential to cause cancer. Persistent infection with any of the known 14 HR-HPV types can result in the development of cervical cancer (International Agency for Research on Cancer, 2022; Wei et al., 2024; see Table 1).

**Table 1.** Over 200 types of HPV have been characterized, 40 of which are associated with genital tract infections. Fourteen are classified as high-risk as persistent infections can lead to cervical cancer, while other low-risk types are associated with genital warts

Category	HPV type
High-risk	16, 18, 31, 33, 35, 39, 45, 51, 52, 56, 58, 59, 66, and 68
Low-risk	6 and 11 ^a^

a These are the most common.

Carcinogenicity varies by genotype with HR-HPV types 16 and 18 contributing to 70% of all cervical cancers, and a further 6 HR types (31, 33, 35, 45, 52 and 58) contributing to an additional 10% of all cervical cancers (International Agency for Research on Cancer, 2022; Wei et al., 2024). There is some geographic variation in the prevalence of different HR-HPV types. For example, HPV 45, 35 and 52 were found to be more common in African women diagnosed with cervical cancer than European, North American, or Chinese women (Wang et al., 2024), although HPV 16 and 18 remained responsible for the majority of cervical cancer.

SSA has the highest HR-HPV prevalence globally at 24% (Bruni et al., 2022) and the highest global burden of cervical cancer (Singh et al., 2023). Moreover, cervical cancer is the leading cause of cancer death in sub-Saharan African women (Sung et al., 2021).

HR-HPV prevalence has been linked to sociological factors including stigma regarding women’s health (Kutz et al., 2023a) and high human immunodeficiency virus (HIV) prevalence (Clifford et al., 2016). Difficulties accessing the HPV vaccine, difficulties accessing condoms, and a lack of sexual health education also contribute to high HR-HPV prevalences (Tchouaket et al., 2023).

**Figure 2.**
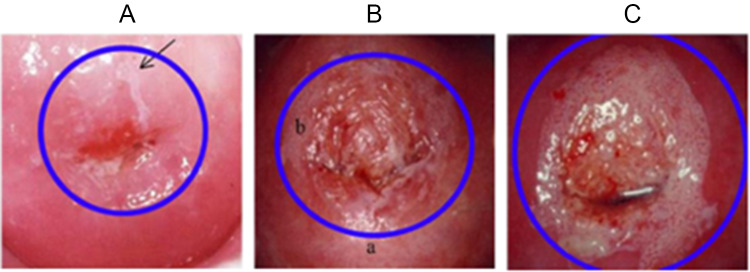
Cervixes with progressively advanced stages of cervical intraepithelial neoplasia (CIN), associated with HR-HPV and the precursor to cervical cancer. The blue ring highlights where pathology is concentrated. (A) CIN 1; (B) CIN 2; and (C) CIN 3. (Figure adapted from Sellor. JW and Sankaranarayanan. R, Colposcopy and Treatment of Cervical Intraepithelial Neoplasia: A Beginner’s Manual, Sellors, 2003).

HR-HPV infection often first presents as cervical intraepithelial neoplasia (CIN), abnormal changes in the cervical cells (see Figure 2). CIN is currently graded on a scale of 1–3, measured by the area of the cervix that the abnormal cells cover. Other than the visual cell disruption, CIN can be completely asymptomatic, leading to delays in treatment. This is especially problematic as CIN can develop into cervical cancer.

### Human papillomavirus diagnosis

HPV NAATs allow detection of HR-HPV infections before cervical precancer develops. Routine cervical cancer screening using HR-HPV NAATs, visual inspection or cervical cytology followed by treatment of precancerous lesions where necessary is highly effective at preventing cervical cancer (World Health Organisation, 2021). It is recommended for women aged 30 years and over for the general population, and 25 years and over for those with HIV (World Health Organisation, 2021). Visual inspection using acetic acid (VIA), is the most common diagnostic method in low resource settings, but the method lacks sensitivity and specificity to detect cervical precancer (Arbyn et al., 2021).

HR-HPV NAATs are recommended in primary screening over VIA or cytology because it has higher sensitivity and specificity to detect cervical precancer risk. Both clinician-collected and self-collected cervico-vaginal swabs can be used for HPV detection (Arbyn et al., 2021).

### Human papillomavirus and cervical cancer prevention

An important facet of HPV control is vaccination, aimed at reducing HR-HPV infections and so reducing the risk of HPV-related cervical cancer. Globally, the uptake of the HPV vaccine remains lower than the WHO target of 90% of 15-year-old girls (Han et al., 2025). Vaccine uptake is lowest regionally in SSA, with a pooled uptake of only 28.53% (Bruni et al., 2022).

Disparities remain in screening coverage (screened at least once in a lifetime) between high-income countries, with average screening coverage of 88%, and low-income countries at only 15% (Bruni et al., 2022). This low coverage is exacerbated in SSA countries due to a lack of organized cervical cancer screening programmes (Anaman-Torgbor et al., 2020).

## Molecular diagnostics

NAATs for FGS and HPV enable sensitive detection of *S. haematobium* and HPV DNA in clinical samples, with greater accuracy over traditional methods and supporting early diagnosis, surveillance, and targeted interventions. PCR, LAMP and RPA/RAA are the main technologies used for NAATs, with PCR requiring thermal cycling, while LAMP and RPA/RAA operate at constant temperatures (isothermal). The main advantage of isothermal methods is their speed, simplicity, and minimal equipment needs, making them well suited for field diagnostics and low-resource settings. The following sections review the molecular targets for FGS and HPV, developed RPA/RAA assays and opportunities for and integrated diagnostic approach.

## S. haematobium *molecular diagnostic DNA regions*

The genome of *S. haematobium* is large and complex (385 Mb) and has not been readily explored in terms of novel diagnostic markers. Current NAATs have focused on a handful of molecular markers, namely the second internal transcribed spacer (ITS-2) of the ribosomal DNA (rDNA) subunit, the Dra1 repeat region, mt COX-1 gene regions, and the rDNA intergenic spacer (IGS). The ITS-2 and the Dra1 repeat regions are the most used regions due to their high sensitivity. Both targets are commonly used in PCR-based assays for detecting *S. haematobium* eggs and DNA in urine and in genital samples (Kjetland et al., 2009; Keller et al., 2020). Hence, the ITS-2 PCR is commonly used as the reference test for comparative diagnostic studies including FGS investigations (Archer et al., 2022; van Bergen et al., 2024).

The commonly used ITS-2 qPCR is a highly sensitive and specific assay targeting a 77-bp fragment but has the limitation that it cannot differentiate between *S. haematobium* and *S. mansoni* DNA. The Dra1 is a 121-bp non-coding repeat sequence that makes up approximately 15% of the *S. haematobium* genome (Hamburger et al., 2001). It has been used frequently in *S. haematobium* RPA development (Rosser et al., 2015). The COX-1 gene, which encodes the cytochrome c oxidase subunit 1 protein, is located within the mitochondrial genome. It was first used in a *Schistosoma* species NAAT in 2008 (ten Hove et al., 2008). It has also been utilized in RAA assays (Zhao et al., 2023a). Finally, the ribosomal IGS is a region between the 28S and 18S ribosomal genes that has been extensively used in the development of *S. haematobium* loop-mediated isothermal amplification (LAMP) assays (Gandasegui et al., 2015; Bayoumi et al., 2016). The IGS region contains unique sequence motifs that can be used for differentiation between *Schistosoma* species, which can be useful for epidemiological investigations.

### Human papillomavirus molecular diagnostic DNA regions

**Figure 3.**
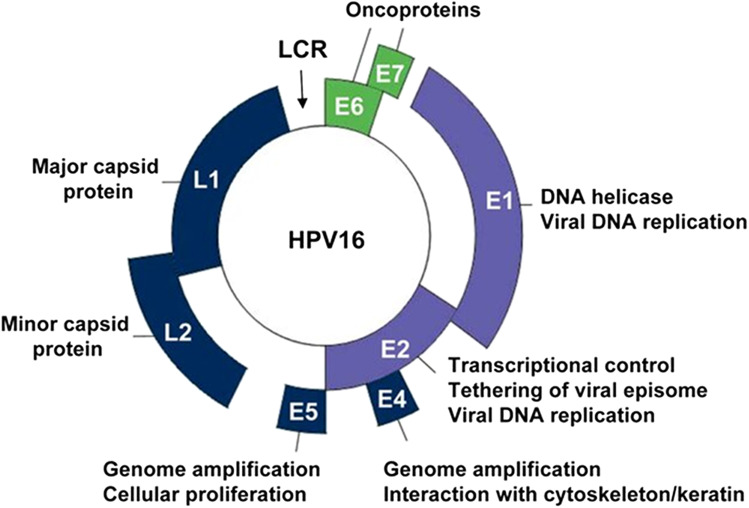
Structure of the HPV genome (figure taken from D’Abramo and Archambault, 2011). The L1 and L2 genes encode for the major and minor capsid proteins respectively. The E6 and E7 genes are oncoproteins.

NAATs for HPV have been in development since the 1980s (Saraiya et al., 2013). At least 264 distinctive commercial molecular tests were available as of December 2023 (Poljak et al., 2024). Despite the high number of available tests, the same target regions of the HPV genome are employed (see Table 2). This is partially due to the HPV genome being relatively small at 7900–8000 kb and having a limited number of genes suitable for molecular diagnostic targets. The HPV genome is divided into 3 major regions – the early gene-coding region (E), the late gene-coding region (L) and the long control region (LCR; see Figure 3).

**Table 2.** Widely used molecular targets in HPV diagnostic assays

Gene name	Genome region	Target function	Conservation between type	Specific diagnostic functions
L1	Late-coding region	Major capsid protein formation	Highly conserved across HPV types	High potential for broad-spectrum testing
L2	Late-coding region	Minor capsid protein formation		
E6	Early-coding region	Targeting and inactivating tumour suppression gene p53	Variability between high-risk HPV types and low-risk HPV types (Oh et al., 2004)	High potential for differential HPV testing
E7	Early-coding region	Disruption of cell-regulating retinoblastoma protein		

L1 and L2 are not expressed at a consistent level throughout the virus lifecycle, with expression being highest in the final stage, during virion formation (Kirk and Graham, 2024). E6 and E7 are known as oncoproteins as their activity is responsible for cells becoming oncogenic as a by-product of the virus hijacking the cell molecular machinery. When E6 and E7 are rendered inactive, oncogenic cells senesce or undergo apoptosis (Yamato et al., 2008; Jabbar et al., 2009).

Due to the strong connection of E6 and E7 gene expression with oncogenicity, with expression increasing as infection develops into cervical precancer and then cervical cancer (Pal and Kundu, 2020), these genome regions are often used for HR-HPV detection. Additionally, as the E6/E7 regions are linked with disease progression, these regions can be used to identify patients at risk of cervical precancer or cancer in quantitative assays (Zhang et al., 2024).

## Recombinase polymerase amplification/recombinase aided amplification diagnostic assays

Isothermal assays, the most common being either LAMP or RPA, have high potential in low-resource settings due to their reduced energy needs and faster run times. LAMP utilizes 4–6 primers per target and so has a more complex primer design in comparison to PCR and RPA/RAA. Although LAMP assays for both *S. haematobium* (van Bergen et al., 2024) and HR-HPV (Fan et al., 2020) have been successfully developed, the complex primer design and relatively high rate of false positives due to primer dimerization (Gruenberg et al., 2018) make it challenging to utilize as a multiplex diagnostic platform. Like PCR, RPA assays have 2 primers per target, making the development and usage of diagnostic assays in low-resource settings, and as a multiplex assay, more feasible.

## S. haematobium *recombinase polymerase amplification/recombinase aided amplification assays*

The first *S. haematobium* RPA assay (Sh-RPA) was based on the Dra1 repeat target due to its potential for high sensitivity (Rosser et al., 2015; see Table 3 for *S. haematobium* RPA assays). Analytical testing using spiked samples reached a limit of detection (LoD) of 100fg of *S. haematobium* gDNA with reactions taking 10 min (Rosser et al., 2015) and amplicon detection via lateral flow strips.

**Table 3.** Published Recombinase Polymerase Amplification/Recombinase Aided Amplification assays for the detection of *S. Haematobium* (S.H) for the diagnosis of urinary and female genital schistosomiasis

Target	Sample			Detection	Analytical		Clinical		Ref. test	Amplification parameters	Ref.
	Type	No.	Prep.		Se. (LoD)	Sp.	Se.	Sp.			
Dra1	*S.h* gDNA spiked urine	NI	NI	LF	100 fg gDNA	*Sch. spp*	NI	NI	NI	30−45 °C/10 min	Rosser et al., 2015
Dra1	Urine (Pemba, Tanzania)	20	DNA kit^1a^	Fluorescence	1 fg gDNA	NI	90%	NI	UEM	40 °C/20 min, portable fluorometer	Rostron et al., 2019
Dra1	Urine (Pemba, Tanzania)	168	DNA kit^1a^	Fluorescence	NI	NI	93.7%	100%	UEM	42 °C/20 min, portable fluorometer	Archer et al., 2020
Dra1	Urine (Kumasi, Ghana)	135	Boiling and DNA kit^1a^	Fluorescence	15 copies	*Sch. spp*	98.4%	100%	Dra 1 qPCR	42°C/15 min	Frimpong et al., 2021
Dra1	VSS, CVL (Zambia)	603, 527	DNA kits ^1a, 2, 3^	Fluorescence	NI	NI	93.3% VSS, 71.4−85.6% CVL	>91%	ITS qPCR	42 °C/20 min, portable flurometer	Archer et al., 2022
Dra1	Syn. *Sh* DNA spiked urine, *Sh* eggs	NI	DNA kit^1b, 5^	Fluorescence	10 fg gDNA	*Sch. spp*	NI	NI	NI	37 °C/40 min	Cherkaoui et al., 2023^b^
Cox1	Syn. DNA, Urine (Zanzibar, Tanzania)	57	DNA kit^5^	Fluorescence	10 copies	*Sh*	NI	NI	UEM	39 °C/20 min	Zhao et al., 2023a
Dra1	Syn. *Sh* spiked DNA, *Sh* eggs, urine (Pemba, Tanzania)	6	DNA kit^1a, 1b, 2, 4^	Fluorescence	≥10 copies, 1 egg	NI	100%	100%	ITS qPCR	42 °C/20 mins	Donnelly et al., 2024^a^
Dra1	Eggs, gDNA		DNA xtraction kit^1b, 4^	Fluorescence and visual using blue-light	10 fg gDNA, 1 egg	*Sch. spp*	NI	NI	ITS qPCR	42 °C f/ 20 min	Mesquita et al., 2025

1a. Speed Extract Kit (Qiagen)/SpeedXtract Nucleid Acid Kit (Qiagen); 1b. SwiftX DNA (Xpedite); 2. Extracta DNA Tissue Prep Kit (Quantabio); 3. QIAamp Mini kit (Qiagen); 4. Dneasy Blood and Tissue Kit (Qiagen); 5. Axyprep™ Body Fluid Viral DNA/RNA Miniprep Kit (Axygen Scientific); 6. Viral genomic DNA Extraction Kit (Bioteke); 7. Maxwell RSCVR ccfDNA plasma kit (Promega); 8. Automated extraction machine; and 9. Reverse blot hybridisation-based assay

a Included the use of Betaine to reduce primer dimer formation.

b Included the use of CRISPR-Cas12 for enhanced sensitivity and specificity.

No, number; Prep., preparation; Se., sensitivity; LoD, limit of detection; Sp., specificity; Ref., reference;

LF, lateral flow; Sch. Spp, *Schistosoma* species; NI, not included; Syn., synthetic;

UEM, urine egg microscopy.

The Sh-RPA continued to be developed into a fluorescence-based assay (Rostron et al., 2019) with amplification detected using a small portable fluorometer. Analytical testing showed a sensitivity of 1fg of DNA. Additionally, 20 frozen urine samples from *S. haematobium* positive individuals from Zanzibar were also tested with 100% sensitivity obtained.

Archer *et al.* further tested Sh-RPA on 168 frozen urine samples with an overall sensitivity and specificity of 93.7% and 100%, respectively, compared to urinary eggs microscopy (Archer et al., 2020). As expected, sensitivity depended on the intensity of infection (eggs/10 mL of urine). Of 168 samples, 73% were classed as having a low or ultra-low egg count (10–49 eggs/10 mL urine and 1–9 eggs/10 mL urine respectively). Of the 70 samples with ultra-low egg counts, 6 were false-negative by RPA, giving a sensitivity of 91.4%. While this was still a high sensitivity, difficulties in detecting ultralow infections may be problematic in elimination settings or areas that have recently received praziquantel MDA. Importantly, the Sh-RPA reactions could be run using a portable fluorometer with a reaction time of 42 °C for 20 min.

Following the development of Sh-RPA for urinary schistosomiasis, the assay was then adapted for detection of *S. haematobium* DNA and/or eggs in FGS samples, namely CVL and cervicovaginal swabs (Archer et al., 2022). Sensitivity ranged between sample types – 93.3% for vaginal self-swab samples (VSS) and 71.4–85.6% for CVL when qPCR was used as the reference test. This was expected as CVL samples have a high volume, resulting in a diluted sample compared to a swab.

In addition to the assay itself, testing was done to identify low-resource extraction methods that maximize clinical sensitivity and specificity (Archer et al., 2022; Donnelly et al., 2024). Archer et al.’s reported the highest sensitivity when VSS and CVL were processed with the now discontinued SpeedXtract Nucleic Acid Kit (Qiagen, Germany), although it has been superseded by the SwiftX DNA kit (Xpedite Diagnostics, Germany). While Donnelly et al. used urine samples, further research showed no significant difference between the SpeedXtract Nucleic Acid Kit and the SwiftX DNA kit in terms of reproducibility between replicates (Donnelly et al., 2024). These are important findings as ensuring a diagnostic assay is resilient to changes in sample preparation methods is important to maintain accessibility and reproducibility of results between laboratories. Clinical sample preparation methodologies are also directly linked to the clinical sensitivity of an assay and should be considered as a vital component of the diagnostic platform.

The Sh-RPA was further refined by Mesquita et al. where the standard DraI RPA oligonucleotides were modified with an aim of improving assay sensitivity and specificity (Mesquita et al., 2025). This included inverting the probe fluorophore and quencher and adding a phosphorothioate backbone to prevent formations of secondary structures, which result in false positives. A comparable sensitivity of 10fg of gDNA was achieved together with improved analytical specificity. Additionally, the storage resilience of the RPA reagents was tested, and they were found to remain stable when stored in the absence of light at ±27 °C for up to 30 days. This supports their portability to, and use in, low-resource settings where cold chains are not readily accessible.

The Dra1 region is the most tested and used target in Sh-RPA assays for both urinary schistosomiasis and FGS, but a mt COX-1 target has also been explored (Zhao et al., 2023a). Although early in development, the assay reached a low LoD of 10 copies in a run time of 20 min at 42 °C and showed a clinical sensitivity of 100% for urine samples, with urine egg microscopy used as the reference test.

### Human papillomavirus polymerase amplification/ assays

Similarly to FGS, RPA/RAA is being explored as suitable low-resource HPV diagnostic platform (see Table 4 for HR HPV RPA assays). However, as HPV is a global pathogen prevalent in high-resource areas, there is less research focus into the development of low-resource NAATs in comparison to a disease such as schistosomiasis.

**Table 4.** Published Recombinase Polymerase Amplification/Recombinase Aided Amplification assays for high-risk human papillomavirus with the purpose of risk assessment for cervical cancer. Adapted from (Ying et al., 2023)

HPV		Sample			Detection	Analytical		Clinical		Ref. test	Am. parameters	Additional technologies	Ref.
Types	Target	Type	No.	Prep.		Se. (LoD)	Sp.	Se.	Sp.				
16, 18	L1	CS (Wenzhou, China)	335	DNA kit^a^	GE, RDB, SYBR Green	0.1 fg/μL (10 Copies/μL)	NI	97.6 − 98.5%	100%	qPCR	37 °C/20 min RPA amplification		Ma et al., 2017
6, 11, 16, 18, 26, 31, 33, 35, 39, 40, 42, 43, 44, 45, 51, 52, 53, 56, 58, 59, 66, 68, 73, 81, 83	L1	CS (Wenzhou, China)	450	Lysis buffer and heating	LF, RDB	100 fg (100 copies) for LFD and 1,000 fg (1,000 copies) for RDB	100%	NI	NI	NI	37 °C/30 min RPA-LFD, 51 °C/30 min RDB		Ma et al., 2019
16, 18	L1	Unspecified clinical samples (Guiyang, China)	89	DNA kit^b^	LF	10 copies (HPV16 and HPV18)	NI	100%	100%	Reverse line blot hybridization	38 °C/30 min	Assisted by graphene oxide and self-avoiding molecular recognition systems	Wang et al., 2020
16, 18	E7	Serum	73	DNA kit^c^	LF	10 copies (HPV16), 5 copies (HPV18)	NI	100%	88.24%	Droplet digital PCR	37 °C/30 min RPA, 7 min visualization		Rungkamoltip et al., 2021
16, 18	L1	CC (Hubei, China)	20	Heating 95 °C/10 min	Bright-field images and fluorescent images	1 copy	NI	92.3%	100%	PCR	39 °C/20 min RPA, 37°C/25 min Cas12a cleavage	CRISPR-Cas12a	Zhao et al., 2023b
16, 18	L1	CC (Hubei, China)	32	95°C/10 min	LF	1 copy	NI	100%	100%		39 °C/12 min RPA, 37 °C/12 min Cas12a cleavage	Cas12 assisted by a heating-membrane- multiplexed microfluidics platform	Zhou et al., 2023
16, 18, 26, 31, 33, 34, 35, 39, 45, 51, 52, 53, 56, 58, 59, 66, 68, 69, 73, 82, 6, 11, 32, 40, 42, 43, 44, 54, 61, 70, 72, 81, 84 and 87	E6, E7, L1	CS (Bangkok, Thailand)	130	Cobas 4800 system^e^	GE	100 − 1000 copies	100%	75%	100%	REBA HPV-ID test^f^	39 °C/40 min, 80 °C/2 min		Wongsamart et al., 2023
16, 18	L1, E7	CS, CB (Shanghai, China)	42 and 36	Phenol-chloroform	Fluorescence	100 − 10,000 copies	100%	88.9%	100%	qPCR	37 °C/25 min		Ying et al., 2023
16, 18, 45	E7	HPV-positive cell lines			LF	50 copies (HPV 16 and 18), 500 copies (HPV 45)	NI	NI	NI	NI	NI	Tailed-primer RPA	Chang et al., 2023
16/18	L1	CS (Wuhan, China)	60	Lysis buffer, incubation 90°C/10 min	Fluorescence	10^−18^ M	100^e^	100%	95.8%	NI	39 °C/15 min	CRISPR/Cas12a, DROPT system	Cai et al., 2024
16	E6/E7	Plasmid DNA, CT (Khon Kaen, Thailand)	40	DNA kit	Electrochemical DNA biosensor	0.23 copies	100%	NI	100%	Electrochemical detection and nested PCR	39°C/20 min		Nakowong et al., 2024

a Viral genomic DNA Extraction Kit (Bioteke). ^b^QIAamp DNAminikits (Qiagen). ^c^Maxwell RSCVR ccfDNA plasma kit (Promega). ^d^QIAamp Viral DNA.

e Automated extraction machine.

f Reverse blot hybridization-based assay.

No, number; Prep., preparation; Se., sensitivity; LoD, limit of detection; Sp., specificity; Ref., reference; CS, cervical swab; CB, cervical biopsy; CT, cervical tissue; NI, not included; Syn., synthetic; LF, lateral flow device; RDB, reverse dot blot; GE, gel electrophoresis.

Due to high number of HPV types, there has been significant development in multiplex RPA assays for HR-HPV. NAATs testing for 34 types, defined in the paper as 20 HR and 14 LR, simultaneously have been developed (Wongsamart et al., 2023). This level of multiplexing was achieved through the innovative detection of both E6/E7 and L1. Both targets had one forward primer, but the L1 target had 2 reverse primers and the E6/E7 target 4 reverse primers, allowing for the selection of different types. The assay proved specific and did not cross react with common bacteria, viruses, and mammalian cells. However, a sensitivity of only 75% for DNA detection all 34 HPV types was achieved, which may have been a result of the primer design which allowed for high levels of multiplexing. The target product profile (TPP) of HPV DNA molecular diagnostics required by the WHO requires a sensitivity of 90–98% for CIN2+ or greater (World Health Organisation, 2024).

Most HR-HPV RPA NAATs target fewer types with a focus on the most oncogenic, typically HPV 16, 18 and occasionally 45. They also focus on achieving higher sensitivity and applicability to low-resource settings. An RPA assay targeting HPV 16 and 18 individually achieved a clinical sensitivity of 97.6–98.5% and specificity of 100% when tested on 335 liquid-based cytology samples extracted using a commercial extraction kit (Viral genomic DNA Extraction Kit) (Bioteke, China) (Ma et al., 2017). Ma *et al.* continued development, multiplexing the assay to detect 25 total HPV types – 12 HR and 13 LR. The assay was tested using plasmids containing the cloned HPV target sequences prepared using a simplified DNA extraction method that involved the addition of a lysis buffer and heating (Ma et al., 2019). The analytical sensitivity was lower in the 2019 assay, at a limit of 10^2^/μL−1 of gDNA, than in the 2017 assay at 0.1 fg/μL (Ma et al., 2017, 2019). This reduction in analytical sensitivity is often seen in NAATs with high levels of multiplexing. This should be kept in consideration as higher sensitivity for HR-HPV types only may be more beneficial clinically than lower sensitivity for a broader range of HPV types.

CRISPR technology has been extensively used alongside RPA assays in HPV diagnostic assays (Tsou et al., 2019;  Gong et al., 2021; Ying et al., 2023) with an aim of increasing sensitivity and specificity. Cai et al.’s use of a CRISPR/Cas12a dual-chamber ‘one-pot’ (DROPT) system, targeting HPV 16/18 had both high sensitivity at 100% and specificity of 95.8% when compared against PCR (Cai et al., 2024). This shows concordance between the DROPT system and PCR. The DROPT system is a one-pot test, and so does not require other large laboratory equipment, which would make it more suitable for field diagnostics. However, issues regarding reliable cold-chain transportation and the extensive extraction process needed for CRISPR Cas technologies means assays utilizing them remain generally unsuitable for low-resource diagnostics.

Alternative assay designs, such as the use of tailed primers (Chang et al., 2023), may increase suitability as they allow for lateral flow devices (LFD), a low-resource assay output method, to be used. An analytical sensitivity of 50 copies/reaction for HPV 16 and 18, and 500 copies/reaction for HPV 45 was achieved. However, as clinical sensitivity and specificity was not investigated, further testing is needed before its suitability as a low-resource diagnostic can be assessed.

The high sensitivity and specificity of HR-HPV RPA/RAA assays in addition to the multiplexing demonstrated shows that the platform has potential to be used as a multiplex diagnostic in clinical settings.

### Utility of potential multiplex diagnostics for female genital schistosomiasis and human papillomavirus

FGS and HR-HPV are common in low-resource areas of SSA, and HR HPV presents a high risk of developing cervical cancer. Additionally, as both FGS and HR-HPV cause physical disruption in the cervix and vaginal canal, making invasion by other pathogens easier, it is hypothesized that having FGS or HR-HPV will increase the risk of acquiring STIs. Relationships have been observed between urogenital schistosomiasis and HIV (Patel et al., 2021) and between HR-HPV and HIV (Okoye et al., 2021). In relation to associations between FGS and HR-HPV, data showed no relationship between FGS and HR-HPV infections in Madagascar (Kutz et al., 2023b), although an association was found between FGS and non-specified LR and HR-HPV types in a South African study (Shukla et al., 2023). Additionally, an association between FGS and HPV 16, 18, and 45 was reported in a recent study conducted in Zambia (Lamberti, 2025). A recent literature review found that data was too limited to confirm or deny a causative connection between FGS and HR-HPV infection (Sturt et al., 2025), in part due to sample size and data quality challenges within individual studies. However, even without a direct association, this does not limit the burden FGS and HR-HPV has on women’s health in SSA.

Multiplex NAATs which detect either *Schistosoma* spp. or HR-HPV with other sample-related pathogens have been developed. For example, a multiplex PCR assay that detects *S. haematobium* and *S. mansoni* in stool samples reported a detection rate of 84.1% in comparison to stool microscopy at 79.5% (ten Hove et al., 2008) and a probe-based LAMP assay for *S. mansoni* and *Strongyloides* spp., a soil-transmitted helminth, has been optimized to work at room temperature for field settings (Crego-Vicente et al., 2023).

As described, extensive multiplex NAATs for different combinations of HPV types have been developed across different molecular platforms. Additionally, multiplex assays that combine different HPV types with other pathogens that can be found in the same sample types, such as *Chlamydia trachomatis, Mycoplasma hominis, Mycoplasma genitalium, Ureaplasma urealyticum* and *Neisseria gonorrhoeae* simultaneously have been developed (Lima et al., 2018).

These studies suggest that the development of a multiplexed NAAT that can detect *S. haematobium* and HR-HPV simultaneously is feasible using different molecular diagnostic platforms and moreover, this has the potential to address a current gap in women’s health screening in SSA.

Currently, no studies have evaluated a combined diagnostic for FGS and HR-HPV. A multiplex molecular diagnostic for FGS and HR-HPV would be beneficial in terms of delivering diagnostic information, but there may be logistical challenges in its implementation in resource-limited settings. Logistical challenges such as supply chain management can result in delays in test-delivery, which is especially pertinent to NAATs which often require cold storage (Kuupiel et al., 2019). Additionally, the initial training needed to familiarize medical personnel, who may not have received molecular biology training before, with new technologies will require significant time and funding (Mfuh et al., 2023).

## Conclusion

FGS and HR-HPV remain significant challenges to women’s health in SSA. Bringing together both HR-HPV and FGS within scalable molecular diagnostic platforms is a step forward towards the integrated screening for FGS and HR-HPV in sub-Saharan Africa. The commonalities of clinical samples used for FGS and HR-HPV screening provide a platform for an integrated screening approach, reducing costs and improving efficiency both for the test provider and the women accessing the service. Continued research and development have led to a range of isothermal diagnostic assays, which are more suited to low-resource settings, being developed for each individually. Sensitive and specific RPA assays exist for both FGS and HR-HPV and so provide an opportunity for the development of a multiplex RPA diagnostic.

There is a need for further research to explore existing molecular targets and technologies that could be combined and used for the development of a low-resource multiplex diagnostic, while keeping economic feasibility in consideration through interdisciplinary research. This includes cost-benefit analysis, field-evaluation, and synergy with existing diagnostic programmes in target countries. Research focus needs to remain on affordability and efficiently providing diagnoses to women in low-resource areas, especially regarding gynaecological health which can be highly stigmatizing. A combined FGS-HR-HPV diagnostic would benefit women living in SSA, allowing women improved FGS-HPV detection and management.

## Notes

**Table of Abbreviations**
CAA: Circulating anodic antigen
CIN: Cervical intraepithelial neoplasia
CVL: Cervicovaginal lavage
FGS: Female genital Schistosomiasis
HIV: Human immunodeficiency virus
HPV: Human papillomavirus
HR-HPV: High-Risk human papillomavirus
IGS: Intergenic spacer
ITS−2: Second internal transcribed spacer
LAMP: Loop-mediated Isothermal Amplification
LCR: Long control region
LoD: Limit of detection
LFD: Lateral flow device
MDA: Mass drug administration
NAAT: Nucleic acid amplification test
NTD: Neglected tropical disease
PCR: Polymerase chain reaction
RAA: Recombinase aided amplification
RPA: Recombinase polymerase amplification
SSA: Sub-Saharan Africa
STI: Sexually transmitted infection
TPP: Target product profile
VIA: Visual inspection with acetic acid
VSS: Vaginal self-swabs
